# Overlapping Distribution of Orexin and Endocannabinoid Receptors and Their Functional Interaction in the Brain of Adult Zebrafish

**DOI:** 10.3389/fnana.2018.00062

**Published:** 2018-07-30

**Authors:** Roberta Imperatore, Livia D’Angelo, Omid Safari, Hamidreza Ahmadniaye Motlagh, Fabiana Piscitelli, Paolo de Girolamo, Luigia Cristino, Ettore Varricchio, Vincenzo di Marzo, Marina Paolucci

**Affiliations:** ^1^Department of Science and Technology (DST), University of Sannio, Benevento, Italy; ^2^Endocannabinoid Research Group, Institute of Biomolecular Chemistry, Pozzuoli, Italy; ^3^Department of Veterinary Medicine and Animal Productions, University of Naples Federico II, Naples, Italy; ^4^Stazione Zoologica Anton Dohrn, Naples, Italy; ^5^Department of Fisheries, Faculty of Natural Resources and Environment, Ferdowsi University of Mashhad, Mashhad, Iran

**Keywords:** orexin/hypocretin receptor OX-2R, cannabinoid receptor (CB1R), confocal microscopy, endocannabinoid levels, zebrafish brain

## Abstract

Hypocretins/Orexins neuropeptides are known to regulate numerous physiological functions, such as energy homeostasis, food intake, sleep/wake cycle, arousal and wakefulness, in vertebrates. Previous studies on mice have revealed an intriguing orexins/endocannabinoids (ECs) signaling interaction at both structural and functional levels, with OX-A behaving as a strong enhancer of 2-arachydonoyl-glycerol (2-AG) biosynthesis. In this study, we describe, for the first time in the brain of zebrafish, the anatomical distribution and co-expression of orexin (OX-2R) and endocannabinoid (CB1R) receptors, suggesting a functional interaction. The immunohistochemical colocalization of these receptors by confocal imaging in the dorsal and ventral telencephalon, suprachiasmatic nucleus (SC), thalamus, hypothalamus, preoptic area (PO) and cerebellum, is reported. Moreover, biochemical quantification of 2-AG levels by LC-MS supports the occurrence of OX-A-induced 2-AG biosynthesis in the zebrafish brain after 3 h of OX-A intraperitoneal (i.p.; 3 pmol/g) or intracerebroventricular (i.c.v.; 0.3 pmol/g) injection. This effect is likely mediated by OX-2R as it is counteracted by i.p./i.c.v administration of OX-2R antagonist (SB334867, 10 pmol/g). This study provides compelling morphological and functional evidence of an OX-2R/CB1R signaling interaction in the brain of adult zebrafish, suggesting the use of this well-established vertebrate animal model for the study of complex and phylogenetically conserved physiological functions.

## Introduction

The high physiological and genetic homology to mammals, as well as the highly conserved anatomical and cytoarchitectural organization have made zebrafish (*Danio rerio*) a very useful animal model in the neuroscience to analyze both brain functions and dysfunctions (Haffter and Nüsslein-Volhard, 1996; Norton and Bally-Cuif, 2010; Wolman and Granato, 2012; Leung et al., 2013; Varshney and Burgess, 2014; D’Angelo et al., 2016b). Neuroanatomical evidence indicates that, for example, components of the zebrafish telencephalon are homologous to the mammalian hippocampus and amygdala, while the midbrain tectum shows similar organization to the cortex of amniotes (Kesavan et al., 2017) or to superior colliculus of mammals (Heap et al., 2018). These highly conserved brain areas are also involved in the control of emotion, learning, memory and movements as in mammals (Wullimann and Mueller, 2004; Broglio et al., 2005; Mueller and Wullimann, 2009; Kesavan et al., 2017).

The orexins are neuropeptides derived from a common precursor, pre-pro-orexin, which is highly conserved in the phylogenetic scale (Sakurai et al., 1998; Shibahara et al., 1999; Galas et al., 2001; Alvarez and Sutcliffe, 2002; Ohkubo et al., 2002; Kaslin et al., 2004; Panula, 2010; Wong et al., 2011). The orexin gene was cloned in zebrafish in 2004 (Kaslin et al., 2004) and it shows a structure very similar to that of mammals, encoding for two active peptides, Orexin-A (OX-A) and Orexin-B (OX-B), homologous to those of mammalian (human, mouse, rat, pig, dog) and nonmammalian (birds, reptiles, amphibians and fish) vertebrates (Sakurai et al., 1998; Shibahara et al., 1999; Hungs et al., 2001; Ohkubo et al., 2002; Kaslin et al., 2004; Faraco et al., 2006; Xu and Volkoff, 2007; Wong et al., 2011). Moreover, a high sequence homology has been found near the C-terminus of OX peptides, which is the region involved in the receptor selectivity and is crucial for orexins biological activity (Darker et al., 2001; Lang et al., 2004; Chiu and Prober, 2013). In the adult zebrafish, as in mammals, the expression of the orexin gene has been found only in a restricted neuronal population of the lateral hypothalamus (LH), which sends projections to most of the brain areas through the telencephalon, diencephalon, mesencephalon and rhombencephalon (Kaslin et al., 2004; Panula et al., 2010; Sundvik et al., 2011; Singh et al., 2015; Sundvik and Panula, 2015). While in mammals the orexins act binding to two specific G protein-coupled receptors, orexin 1 and orexin 2 receptors (OX-1R and OX-2R; de Lecea et al., 1998; Peyron et al., 1998; Sakurai et al., 1998), only one OX receptor, corresponding structurally to the mammalian OX-2R, has been identified in zebrafish (Yokogawa et al., 2007; Wong et al., 2011). The expression of OX-2R has been reported in several brain areas of adult zebrafish, which correspond to the orexinergic neuron projection areas, such as the dorsal and ventral telencephalon, the ventromedial (VM) hypothalamus; the ventral, lateral and caudal hypothalamic nuclei; the caudal zone of the periventricular hypothalamus; the thalamic nuclei; the pretectal nuclei; the periventricular gray zone (PGZ) of optic tectum; the periventricular nucleus of posterior tuberculum; the lateral nuclei in the dorsal tectum and tegmentum, and the locus coeruleus (Kaslin et al., 2004). Several studies have reported that in zebrafish, as in mammals, the orexins signaling is involved in the regulation of many physiological functions, such as sleep/wake cycle, energy homeostasis and locomotor activity (Prober et al., 2006; Panula, 2010; Elbaz et al., 2013; Tsujino and Sakurai, 2013). The overexpression of orexins promotes wakefulness and increases locomotor activity during active state, without affecting the rhythmicity of movements (Prober et al., 2006; Yokogawa et al., 2007). Furthermore, long-term food deprivation leads to a significant increase of pre-pro-orexin mRNA levels in zebrafish and goldfish brain, while intracerebroventricular (i.c.v) injection of OX-A induces feeding, as in mammals (Novak et al., 2005; Nakamachi et al., 2006; Yokobori et al., 2011).

While a massive amount of data has been reported on the endocannabinoid system in mammals, little information exists in fish. As revealed by phylogenetic analyses, the endocannabinoid system is highly conserved between zebrafish and mammals (McPartland et al., 2007; Klee et al., 2012). The endocannabinoids (ECs), mainly the 2-arachydonoyl-glycerol (2-AG), act through the cannabinoid receptor 1 (CB1R), which represents the most plentiful G protein coupled receptor within the central nervous system (Pertwee, 1997). CB1R regulates the majority of cannabinoid central actions (Herkenham et al., 1990; Piomelli, 2003) and numerous biological processes (Herkenham et al., 1990, 1991; Zou and Kumar, 2018). CB1R stimulation affects changes in food intake (Silvestri and Di Marzo, 2013; Krug and Clark, 2015) and promotes obesity, while downregulation of CB1R leads to a reduction of appetite (Osei-Hyiaman et al., 2005; Gary-Bobo et al., 2007; Shimada et al., 2012). CB1R mRNA expression within the zebrafish brain has been detected throughout the telencephalon, pretectum, torus longitudinalis (TL), hypothalamus, tegmentum, anterior rhombencephalon and periventricular hypothalamus, sharing homologies with mammalian CB1R distribution (Lam et al., 2006). CB1R sequence analysis shows a high degree of conservation, especially in those residues critical for ligand recognition and function in mammals (McPartland and Glass, 2003), providing strong support that the zebrafish CB1R is a functional homologous of the human.

Several studies have evidenced the ability of OX-A to potentiate CB1R signaling in mammals. Indeed, the endocannabinoid 2-AG can act as paracrine or autocrine factor leading, respectively, to an OX-1R/CB1R physical interaction and potentiation of CB1R activity, or to a functional regulation of CB1R at presynaptic level and consequent possible modulation of synaptic plasticity. By these physical and functional interactions, OX-1R and CB1R can control many physiological and pathophysiological functions (Cristino et al., 2013, 2016, 2017; Flores et al., 2013; Imperatore et al., 2016; Morello et al., 2016). Very few data are available on CB1R-OX-2R interaction, addressing contrasting evidences on their functional interactions (Yazdi et al., 2015; Esmaeili et al., 2017). The relative simplicity of orexins signaling in the zebrafish brain, due to the low number of OX neurons and the expression of only one OX receptor, and the highly conserved ECs functions, make zebrafish an ideal animal model to study the interaction between orexins and ECs.

In this framework, the aim of our study was to investigate whether in the zebrafish brain OX-2R and CB1R show an overlapping pattern of distribution and functional interaction similar to those suggested in the mouse. These data allow us to propose zebrafish as an animal model for the investigation of the basic and conserved physiological functions controlled by orexins and ECs signaling, highly preserved among the vertebrates. In this study, we therefore employed immunohistochemistry and confocal imaging to define the OX-2R/CB1R receptors anatomical distribution and co-expression in the whole zebrafish brain, as well as the liquid chromatography-atmospheric pressure chemical ionization-mass spectrometry (LC-APCI-MS) to measure the changes of 2-AG levels induced by OX-A, to investigate the regulation of the 2-AG production mediated by OX-2R activation.

## Materials and Methods

### Animals

Animals were housed under standard conditions of photoperiod (14:10 LD; ZT0, 9 AM) and temperature (28°C). The current study was carried out in the Aquaculture Lab of the Ferdowsi University of Mashhad (FUM). This project was approved by FUM animal ethics committee. Fish used in this study were treated in accordance with the European Commission recommendation 2007/526/EC and 2010/63/UE on revised guidelines for the accommodation and care of animals used for experimentation and other scientific purposes. All efforts were made to minimize fish suffering. Zebrafish did not receive medical treatment prior or during the experience. No deaths occurred in the facilities before the euthanasia of animals used for the experiments.

### Tissue Processing and Immunohistochemistry

For the immunohistochemistry, 4-month-old females (*n* = 6) and males (*n* = 6) were suppressed in ice water. After skull opening, brains were removed and fixed overnight 4°C by immersion in phosphate-buffered saline (PBS; pH 7.4) containing 4% paraformaldehyde (PFA). The brains were washed in PBS and transferred to 20% sucrose overnight (ON), followed by 30% sucrose in 0.1 M PBS ON at 4°C. The brains were frozen and embedded in a Frozen Section Media (Leica Biosystems), cryosectioned with a Leica CM3050S cryostat into 12 μm-thick serial sections in the coronal plane, collected in alternate series and processed for immunofluorescence. The sections were washed with 0.25% Triton X-100 in PBS, pH 7.4 (PBS-T), and incubated for 12 h at room temperature with primary antibodies diluted in PBS-T. The following primary antibodies were used: goat antibody against OX-2R diluted 1:200 (code sc-8074; Santa Cruz Biotechnology, Santa Cruz, CA, USA), rabbit antibody against CB1R diluted 1:200 (code ab23703; Abcam, Cambridge, UK). After the incubation with the primary antibodies, the sections were washed several times in PBS-T and incubated for 2 h at room temperature with donkey anti rabbit Alexa 488- and donkey anti goat Alexa 594-conjugated secondary antibodies (1:100; Invitrogen, ThermoFisher Scientific, France). Tissue sections were washed in PBS and finally counterstained with DAPI (4’,6-diamidino-2-phenylindole; Sigma-Aldrich S.r.l., Milan, Italy). All slides were coverslipped with Aquatex mounting medium (Merck, Darmstadt, Germany). The immunostained sections were observed with a confocal microscopy Nikon Eclipse Ti2 (Nikon, Florence, Italy) equipped with x-y-z motorized stage, a digital camera DS-Qi2 (Nikon, Florence, Italy) and the acquisition and Image analysis software NIS-Elements C (Nikon, Florence, Italy). Digital images were acquired using the 5-20-40× objectives. We collected serial Z-stacks of images throughout the area of interest (6–10 planes with an increment varying 0.5–1 μm). Images were deconvolved using the imaging deconvolution software by application of ten iterations. Serial Z planes images were collapsed into a single maximum projection image. Micrographs were saved in TIFF format and adjusted for light and contrast before being assembled on plates using Adobe Photoshop 6.01 (Adobe Systems, San Jose, CA, USA).

### Controls

The specificity of the antibodies was validated with controls that included: (1) omission of primary or secondary antibody staining; (2) pre-absorptions of each primary antibody with an excess of the relative peptide (OX-2R peptide, Santa Cruz; CB1R peptide, Abcam; 100 mg of peptide/ml of diluted antiserum). Internal reaction controls were carried out by substituting primary antisera or secondary antisera with PBS or normal serum in the specific step. A further control of CB1R antibody was done by aligning the epitope sequence and the aminoacid sequence of the protein (Accession: NP_997985.1 GI: 47086397). For OX-2R, we aligned the aminoacids sequences of *Homo sapiens* and *Danio rerio* (Accession: ABO61386.1 GI: 134142085) because the epitope was not available. Alignments were done by using Multiple Sequence Alignment.

### Protein Extraction and Western Blotting

Four months of female (*n* = 3) and male (*n* = 3) zebrafish brain were frozen and homogenized in buffer (consisting of 50 mM Tris–HCl, pH 7.0, 150 mM NaCl, 2% Triton, 5 mM EDTA, 10 mg/ml leupeptin, 0.1 U/ml aprotinin, 1 mM PMSF) using an Ultra-Turrax homogenizer and centrifuged at 16,000 *g* for 20 min at 4°C. Aliquots of supernatant were subjected to SDS–PAGE analysis using 4% to 12% Bis-Trisgels (NuPAGE, Invitrogen). After separation, the proteins were electrophoretically transferred to nitrocellulose membrane with the iBlot transfer system (Invitrogen). Then the membranes were blocked in TBS-T buffer (150 mM NaCl, 20 mM TrisHCl pH 7.4, 0.1% Tween-20) containing 5 g 100 mL-1 milk for 1 h at room temperature. The blots were then incubated ON with primary antibody anti-OX-2R (code sc-8074; Santa Cruz Biotechnology Inc., Santa Cruz, CA USA) produced in goat, diluted 1:500, or anti-CB1R (code ab23703; Abcam, Cambridge, UK) produced in rabbit, diluted 1:100, in TBS-T and containing 2.5% milk. After three washes in TBS-T, the membranes were incubated in TBS-T for 1 h with secondary antibody, horseradish peroxidase conjugated anti-goat (code 305-035-003; 1:1000, Immuno Research) for OX-2R and anti-rabbit (code sc-2004; 1:10,000; Santa Cruz Biotechnology Inc., Santa Cruz, CA USA) for CB1R, respectively. Proteins were visualized with the ECL Advanced Western blotting detection kit (Amersham) on C-Digit System. Homogenate of mouse hypothalamus was employed as positive control.

### Pharmacological Treatment

The animals (*n* = 3 per group) were treated with intraperitoneal (i.p.) or intracerebroventricular (i.c.v.) injection of OX-A (TOCRIS, Bristol, United Kingdom) at the dose of 3 pmol/g or 0.3 pmol/g, respectively (Yokobori et al., 2011). For i.p. and i.c.v. injection, the effective dose of OX-A and SB334867 was obtained by a dose-response curve. For OX-A doses were 0.3, 3 and 30 pmol/g; for SB334867 0.1, 1 and 10 pmol/g. Another group of animals (*n* = 3 per group) was pretreated with i.p. or i.c.v. injection of orexin receptor antagonist SB334867 (10 pmol/g; TOCRIS, Bristol, United Kingdom) followed by OX-A i.p. injection 30 min later or i.c.v. injection 15 min later. SB334867 has been reported to be able to antagonize orexin at the fish orexin receptor (Miura et al., 2007; Yokobori et al., 2011). The treatment was performed at 9–11 AM to avoid any possible circadian variability. The skull was opened and zebrafish brains were rapidly removed after 3 h of i.p and 1 h of i.c.v. treatment. ICV administration was carried out as described previously (Yokobori et al., 2011).

### Lipid Extraction and Measurement of Endocannabinoid Levels in the Brain

Each single brain was homogenized in 5 volume chloroform/methanol/Tris·HCl (50 mmol L–1, pH 7.5; 2:1:1, volume-to-volume ratio) containing 10 pmol of [H]_8_-Anandamide ([H]_8_-AEA) and 50 pmol of [H]_5_-2-arachidonoylglycerol ([H]_5_-2-AG) as internal standards. Homogenates were centrifuged at 13,000× *g* for 16 min (4°C), the aqueous phase plus debris was collected and extracted four times with 1 volume chloroform. The lipid-containing organic phases were dried down, weighed and pre-purified by open-bed chromatography on silica columns eluted with increasing concentrations of methanol in chloroform. Fractions for 2-AG and AEA measurement were obtained by eluting the columns with 9:1 (by volume) chloroform/methanol and then analyzed by liquid chromatography-atmospheric pressure chemical ionization-mass spectrometry (LC-APCI-MS), by using a Shimadzu high-performance liquid chromatography apparatus (LC-10ADVP) coupled to a Shimadzu quadruple mass spectrometer (LCMS-2020) via a Shimadzu atmospheric pressure chemical ionization interface. LC-APCI-MS analyses were carried out in the selected ion monitoring mode, using m/z values of 384.35 and 379.35 (molecular ions +1 for deuterated and undeuterated 2-AG), 356 and 348 (molecular ions +1 for deuterated and undeuterated AEA). The amounts of analyses in tissues quantified by isotope dilution with the above mentioned deuterated standards were expressed as picomoles per milligram of lipid extract.

### Statistical Analyses

The data are expressed as mean ± SEM and were analyzed with GraphPad Prism six software, version 6.05 (GraphPad Inc.). Statistical differences among groups were determined by one-way ANOVA followed by *post hoc* Tukey test for comparison among means. *p* < 0.05 was considered statistically significant.

## Results

### OX-2R and CB1R Co-expression in the Adult Brain

**Figure 1:**
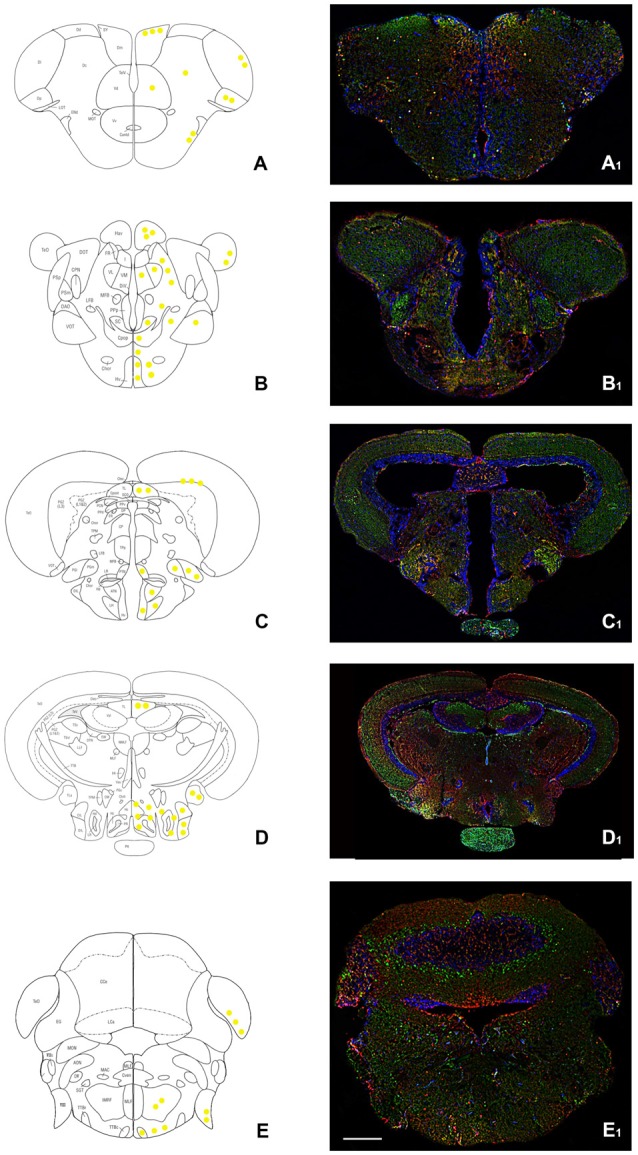
Overview of orexin 2 receptors (OX-2R; red) and cannabinoid receptor 1 (CB1R; green) immunoreactivity in the whole adult zebrafish brain. **(A–E)** Schematic drawings of five coronal sections of the adult zebrafish brain showing, at right, the distribution of OX-2R/CB1R co-expression (yellow solid circles) and, at left, the brain nuclei and regions according to Wullimann et al. (1996). **(A_1_–E_1_)** Photomicrographs of the sections corresponding to the schematic coronal sections reported in **(A–E)**. **(A,A_1_)** Telencephalon: OX-2R/CB1R co-localization in lateral (Dl), medial (Dm), central (Dc) and posterior (Dp) part of the dorsal telencephalon and in the dorsal (Vd) and lateral (Vl) part of the ventral telencephalon. **(B,B_1_)** Diencephalon: OX-2R/CB1R co-localization in ventral habenular nucleus (Hav), optic tectum (TeO), ventral zone of the periventricular hypothalamus (Hv). **(C,C_1_)** Dienchephalon/mesencephalon: OX-2R/CB1R co-localization in the torus longitudinalis (TL), medial (PGm) and lateral (PGl) preglomerular nuclei, lateral hypotalamus (LH), Hv, and anterior (ATN) and posterior (PTN) tuberal nuclei. **(D,D_1_)** Mesencephalon: OX-2R/CB1R co-localization in TL, caudal (Hc) and dorsal (Hd) zone of the periventricular hypothalamus, torus lateralis (Tla), central nucleus of inferior lobe (CIL) and diffuse nucleus of inferior lobe (DIL). **(E,E_1_)** Rhombencephalon: OX-2R/CB1R co-localization in TeO, octaval nerve (VIII), intermediate reticular formation (IMRF) and tractus tectobulbaris (TTB). AON, anterior octaval nucleus; Cantd, commissura anterior, pars dorsalis; Cce, corpus cerebelli; Chor, Commissura horizontalis; CM, corpus mammillaris; CP, central posterior thalamic nucleus; CPN, central pretectal nucelus; Cpop, commissura postoptica; DAO, dorsal accessory optic nucleus; DiV, Diencephalic ventricle; DOT, dorsomedial optic tract; DP, dorsal posterior thalamic nucleus; DTN, dorsal tegmental nucleus; EG, eminentia granularis; End, entopeduncular nucleus, dorsal part; FR, fasciculus retroflexus; I, intermediate thalamic nucleus; Lca, lobus caudalis cerebelli; LFB, lateral forebrain bundle; LLF, lateral longitudinal fascicle; LOT, lateral olfactory tract; MFB, medial forebrain bundle; MLF, medial longitudinal fascicle; MON, medial octavolateralis nucleus; MOT, medial olfactory tract; PGZ, Periventricular gray zone of the optic tectum; Pit, pituitary; PPd, periventricular pretectal nucleus, dorsal part; PPp, parvocellular preoptic nucleus, posterior part; PPv, periventricular pretectal nucleus, ventral part; PSm, magnocellular superficial pretectal nucleus; PSp, parvocellular superficial pretectal nucleus; SC, suprachiasmatic nucleus; SGT, secondary gustatory tract; TelV, telencephalic ventricle; TeV, Tectal ventricle; TPM, tractus pretectomammillaris; TPp, periventricular nucleus of posterior tuberculum; TS, torus semicircularis; Val, lateral division of valvula cerebelli; VL, Ventrolateral thalamic nucleus; VM, Ventromedial thalamic nucleus; VOT, ventral optic tract; Vv, ventral nucleus of ventral telencephalic area. Scale bar, 250 μm.

The analysis of OX-2R/CB1R colocalization was carried out in the whole brain of adult zebrafish. The anatomical description and the nomenclature follow the atlas by Wullimann et al. (1996). An overview of the co-distribution and co-expression of OX-2R and CB1R is presented in the scheme in Figure 1 and Table 1.

**Table 1:** Co-distribution of OX-2R and CB1R in the zebrafish brain and homologous mammalian brain regions.

	Zebrafish brain regions	Mammalian brain regions	Overlapping distributions of OX-2R and CB1R

Telencephalon	Vl	Basal ganglia	+
	Vd	Basal ganglia	+
	Dm	Amygdala	+
	Dd		+
	Dc		+
	Dl	Hippocampus	+
	Dp		+
	NT		+
Diencephalon	VM		+
	VL		+
	Hav	Medial habenular nuclei	+
	Had	Lateral habenular nuclei	+
	CP		+
	Parvocellular preoptic nucleus		+
	Magnocellular nucleus	Paraventricular nucleus	+
	SC		+
	VOT		+
	LFB		+
	Hv	Arcuate nucleus	+
Basal diencephalon/	PTN		+
Mesencephalon	PGl	Sensory thalamic nuclei	+
	PGm	Sensory thalamic nuclei	+
	LH		+
	Hd		+
	Hc		+
	TLa		+
	DIL		+
	TeO	Visual cortex or superior colliculus	+
	TeV		+
	TL		+
	TTB		+
Rhombencephalon	Medulla oblongata	Medulla oblongata	
	IMRF	Reticular formation	+
	VIII		+

The specificity of both OX-2R and CB1 in the zebrafish brain was evaluated by Western blotting (Supplementary Figure S1) and phylogenetic analyses (Supplementary Figure S2) to confirm the degree of conservation. In zebrafish, as in the mouse hypothalamus (used as positive control), the polyclonal antibody against OX-2R showed one band of approximately 38 kDa (OX-2R) and that against CB1R of about 60 kDa, according to what has also been reported in the datasheet of the antibodies (Supplementary Figure S1) and by previous studies on fish (D’Angelo et al., 2016a). In addition, sequences alignment confirmed that zebrafish OX-2R aminoacid sequence share a 72.6% of identity with human (412 aminoacid residues overlap) and the epitope of the CB1R antibody revealed a degree of conservation of 75%, being 9 out of 12 conserved aminoacids (Supplementary Figure S2).

### Telencephalon

**Figure 2:**
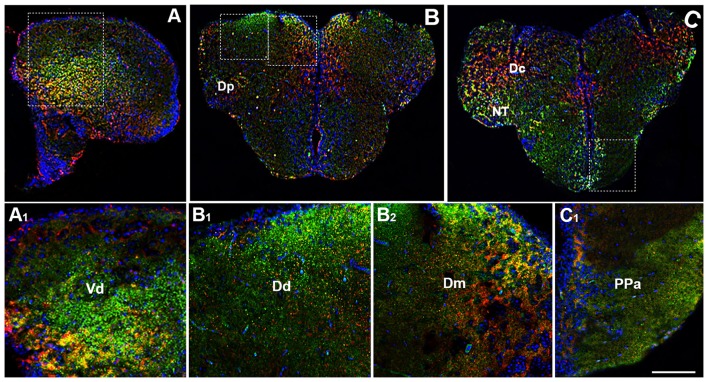
Distribution of OX-2R (red) and CB1R (green), and their co-localization OX-2R/CB1R (yellow) in coronal sections of the telencephalon. **(A–C)** OX-2R/CB1R co-expression has been observed in the dorsal and ventral telencephalon of zebrafish brain. **(A_1_)** Higher magnification of the field boxed in **(A)** showing the OX-2R/CB1R co-expression within the dorsal nucleus of the ventral telencephalon (Vd). **(B_1_,B_2_)** Higher magnification of the fields boxed in **(B)** showing the OX-2R/CB1R co-expression within the dorsal (Dd) **(B_1_)** and medial (Dm) **(B_2_)** part of the dorsal telencephalon. **(C_1_)** Higher magnification of the field boxed in **(C)** showing the OX-2R/CB1R co-expression within the parvocellular preoptic nucleus, anterior part (Ppa). DAPI (Blue) was used as a counterstaining to show nuclei. Dc, central part of the dorsal telencephalon; Dp, posterior part of the dorsal telencephalon; NT, nucleus taeniae. Scale bar, 250 μm for **(A–C)**; 50 μm for **(A_1_,B_1_,B_2_,C_1_)**.

By using immunohistochemistry, we found a co-expression of OX-2R and CB1R in several telencephalic structures. In the brain of adult zebrafish, the telencephalon includes two major subdivisions: the dorsal telencephalon (pallium) and ventral telencephalon (subpallium). Dorsal and ventral telencephalon can be further divided into minor fields depending on the cytoarchitecture and fiber connections (Nieuwenhuys, 2009; Ganz et al., 2014). OX-2R/CB1R co-staining was observed in the lateral nucleus (Vl) and dorsal nucleus (Vd) of the ventral telencephalic area (Figures 2A,A_1_) and in the whole dorsal telencephalic area (D; Figure 1), i.e., in the medial (Dm), dorsal (Dd), central (Dc), lateral (Dl) and posterior (Dp) part of the dorsal telencephalic area (Figures 2B,B_1_,B_2_,C,C_1_). Moreover, OX-2R/CB1R co-localization was found in the nucleus taeniae (NT) and in close contact with the telencephalic ventricle in the parvocellular preoptic nucleus, anterior part (Ppa; Figures 2C,C_1_).

### Diencephalon

The immunohistochemical analysis of adult zebrafish diencephalon showed OX-2R/CB1R co-localization also in this zebrafish brain region. This brain region comprises the prethalamus, habenula and thalamus, which are brain areas highly conserved across vertebrates (Stephenson-Jones et al., 2011; Cheng et al., 2014).

**Figure 3:**
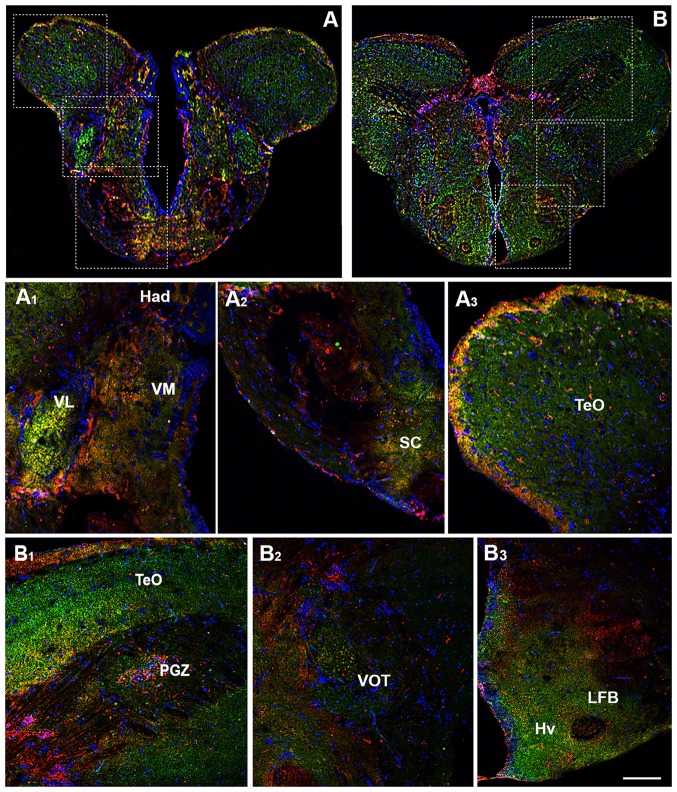
Distribution of OX-2R (red) and CB1R (green), and their co-localization OX-2R/CB1R (yellow) in coronal sections of the diencephalon. **(A,B)** OX-2R/CB1R co-expression has been observed in the suprachiasmatic nucleus (SC), ventrolateral (VL) and ventromedial (VM) thalamic nuclei, dorsal habenular nucleus (Had), optic tectum (TeO), periventricular gray zone of the optic tectum (PGZ), ventral optic tract (VOT), lateral forebrain bundle (LFB) and ventral zone of the periventricular hypothalamus (Hv). **(A_1_–A_3_)** Higher magnification of the fields boxed in **(A)** showing the OX-2R/CB1R co-expression within the Had, VL, VM **(A_1_**), SC **(A_2_)** and TeO **(A_3_)**. **(B_1_–B_3_)** Higher magnification of the fields boxed in **(B)** showing the OX-2R/CB1R co-expression within the TeO, PGZ **(B_1_)**, VOT **(B_2_)**, LFB and Hv **(B_3_)**. DAPI (Blue) was used as a counterstaining to show nuclei. Scale bar, 250 μm for **(A,B)**; 50 μm for **(A_1_–A_3_,B_1_–B_3_)**.

**Figure 4:**
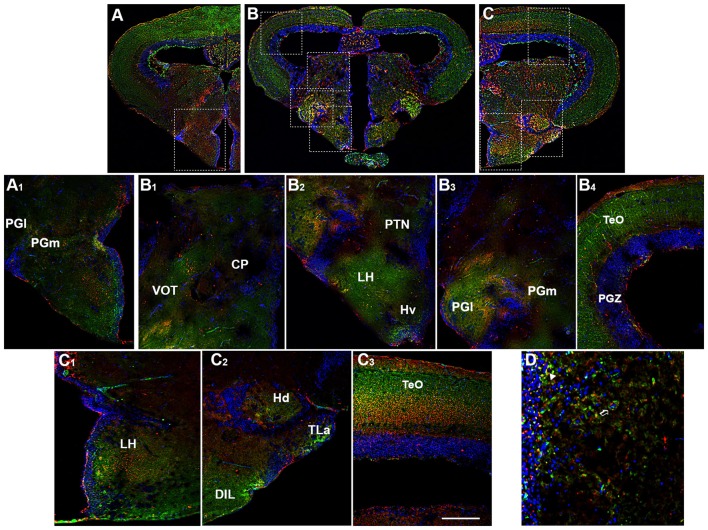
Distribution of OX-2R (red) and CB1R (green), and their co-localization OX-2R/CB1R (yellow) in coronal sections of the diencephalon/midbrain. **(A–C)** OX-2R/CB1R co-expression has been observed in the lateral (PGl) and medial (PGm) preglomerular nuclei, VOT, central posterior thalamic nucleus (CP), posterior tuberal nucleus (PTN), lateral hypothalamus (LH), ventral zone of the periventricular hypothalamus (Hv), periventricular gray zone of the optic tectum (PGZ), optic tectum (TeO), dorsal zone (Hd) of the periventricular hypothalamus, torus lateralis (Tla), diffuse nucleus of inferior lobe (DIL). **(A_1_)** Higher magnification of the field boxed in **(A)** showing the OX-2R/CB1R co-expression within the PGl and PGm. **(B_1_–B_4_)** Higher magnification of the fields boxed in **(B)** showing the OX-2R/CB1R co-expression within the CP and VOT **(B_1_)**, PTN, LH and Hv **(B_2_)**, PGl and PGm **(B_3_)**, TeO and PGZ **(B_4_)**. **(C_1_–C_3_)** Higher magnification of the fields boxed in **(C)** showing the OX-2R/CB1R co-expression within the LH **(C_1_)**, Hd, Tla and DIL **(C_2_)**, TeO **(C_3_)**. **(D)** Detail of OX-2R/CB1R co-expression in LH: arrow showing a putative adjacent localization of OX-2R/CB1R and arrowhead showing a putative co-localization and overlapping of OX-2R/CB1R in the same cells. DAPI (Blue) was used as a counterstaining to show nuclei. Scale bar, 250 μm for **(A–C)**; 50 μm for **(A_1_,B_1_–B_4_,C_1_–C_3_)**; 25 μm for **(D)**.

The prethalamus includes the VM and the ventrolateral (VL) thalamic nuclei which are present in zebrafish, but not in the mammalian brain (Mueller, 2012). Both nuclei showed the OX-2R/CB1R co-expression (Figures 3A,A_1_). The habenula includes the ventral (Hav) and dorsal (Had) habenular nuclei and the OX-2R/CB1R co-expression was found mainly in the Had (Figures 3A,A_1_). The thalamus is subdivided into the anterior thalamic nucleus, the dorsal posterior thalamic nucleus and the central posterior thalamic nucleus (CP). The thalamus, in particular the CP, showed OX-2R/CB1R co-expression (Figures 4B,B_1_).

Another main area of the diencephalon is the preoptic area (PO) which includes different subnuclei, such as the anterior and posterior parvocellular and the magnocellular preoptic nuclei, and the suprachiasmatic nucleus (SC; Braford and Northcutt, 1983; Wullimann et al., 1996; Rupp and Northcutt, 1998; Filippi et al., 2010; Yamamoto et al., 2010, 2011). All of these diencephalic nuclei showed OX-2R/CB1R co-localization (Figures 3A,A_2_).

Moving caudally along the diencephalon, other three regions were characterized by the OX-2R/CB1R co-expression: the ventral optic tract (VOT; Figures 3B,B_2_, 4B,B_1_), the lateral forebrain bundle (LFB; Figures 3B,B_3_) and the ventral zone of the periventricular hypothalamus (Hv; Figures 3B,B_3_, 4B,B_2_).

### Basal Diencephalon/Mesencephalon

The basal diencephalon of zebrafish comprises the posterior tuberculum and the preglomerular complex. The posterior tuberculum includes all the nuclei intercalated between prethalamus and hypothalamus, such as periventricular nucleus of the posterior tuberculum, paraventricular organ and posterior tuberal nucleus (PTN; Rupp et al., 1996; Wullimann et al., 1996; Herget et al., 2014). The preglomerular complex is divided in anterior, lateral, medial and caudal preglomerular nuclei (Pga, PGl, PGm, PGc). The OX-2R/CB1R co-expression was detected in the PTN (Figures 4B,B_2_), PGl and PGm (Figures 4A,A_1_,B,B_3_).

By morphological analysis, OX-2R/CB1R co-localization was found mainly in the following hypothalamic region: LH (Figures 4B,B_2_,C,C_1_,D), dorsal (Hd; Figures 4C,C_2_, 5A,A_1_,B,B_2_, 6A,A_4_) and caudal (Hc) zone of periventricular hypothalamus (5A,A_1_,B,B_3_, 6A,A_5_), torus lateralis (Tla; Figures 4C,C_2_) and diffuse nucleus of inferior lobe (DIL; Figures 4C,C_2_, 5A,A_1_,B,B_2_, 6B,B_1_).

**Figure 5:**
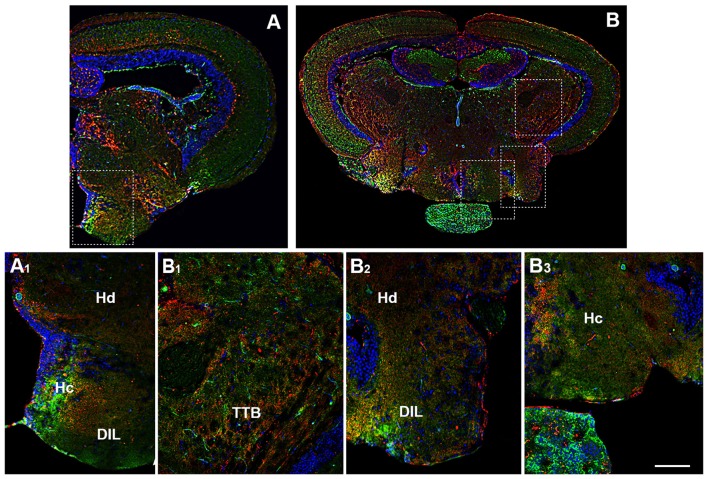
Distribution of OX-2R (red) and CB1R (green), and their co-localization OX-2R/CB1R (yellow) in coronal sections of diencephalon/mesencephalon. **(A,B)** OX-2R/CB1R co-expression has been observed in the dorsal (Hd) and central (Hc) zone of the periventricular hypothalamus, DIL and TTB. **(A_1_)** Higher magnification of the field boxed in **(A)** showing the OX-2R/CB1R co-expression within the Hc, Hd and DIL. **(B_1_–B_3_)** Higher magnification of the fields boxed in **(B)** showing the OX-2R/CB1R co-expression within the TTB **(B_1_)**, Hd and DIL **(B_2_)**, Hc **(B_3_)**. DAPI (Blue) was used as a counterstaining to show nuclei. Scale bar, 250 μm for **(A,B)**; 50 μm for **(A_1_,B_1_–B_3_)**.

**Figure 6:**
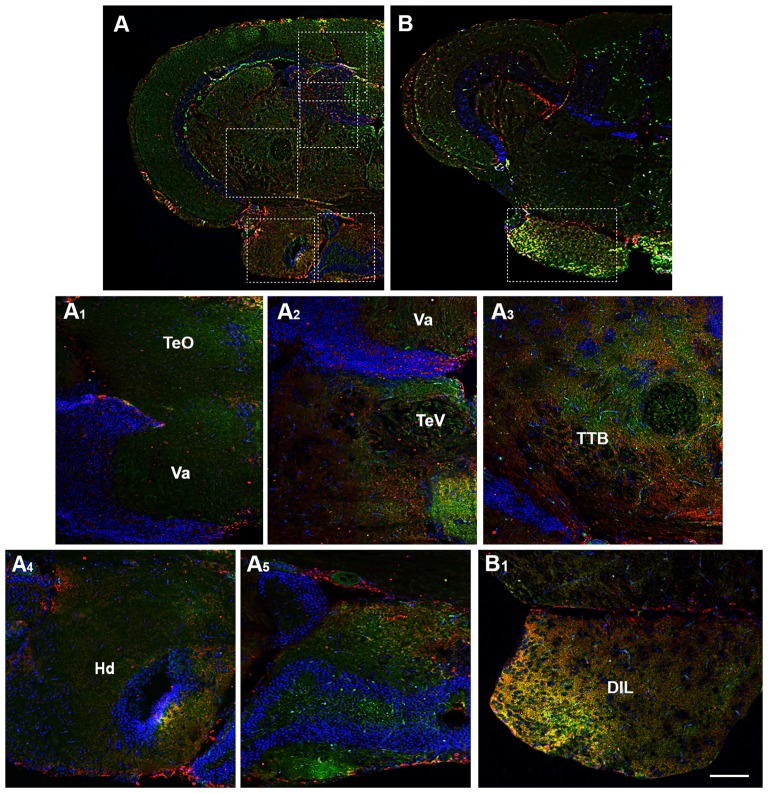
Distribution of OX-2R (red) and CB1R (green), and their co-localization OX-2R/CB1R (yellow) in coronal sections of the midbrain. **(A,B)** OX-2R/CB1R co-expression has been observed in the optic tectum (TeO), along the tectal ventricle (TeV), TTB, dorsal (Hd) and central (Hc) zone of the periventricular hypothalamus, DIL. **(A_1_–A_5_)** Higher magnification of the fields boxed in **(A)** showing the OX-2R/CB1R co-expression within the TeO **(A_1_)**, TeV **(A_2_)**, TTB **(A_3_)**, Hd **(A_4_)**, Hc **(A_5_)**. **(B_1_)** Higher magnification of the field boxed in **(B)** showing the OX-2R/CB1R co-expression within the DIL. DAPI (Blue) was used as a counterstaining to show nuclei. Va, Valvula Cerebelli. Scale bar, 250 μm for **(A,B)**; 50 μm for **(A_1_–A_5_,B_1_)**.

Moving caudally towards the midbrain, prominent structure is the optic tectum (TeO). Immunohistochemical studies evidenced OX-2R/CB1R co-staining in the PGZ of the TeO (Figures 3B,B_1_, 4B,B_1_) and in the central and more external layers of the TeO (Figures 3A,A_3_,B,B_1_, 4B,B_4_,C,C_3_, 6A,A_1_), but also around the tectal ventricle (TeV; Figures 6A,A_2_) and in the TL (Figure 4), which is a conspicuous paired structure of the midbrain connecting the bilateral tectal lobes. Moreover, the morphological analysis showed OX-2R/CB1R co-expression in the midbrain tegmentum, particularly in the tractus tectobulbaris (TTB; Figures 5B,B_1_, 6A,A_3_).

### Rhombencephalon

**Figure 7:**
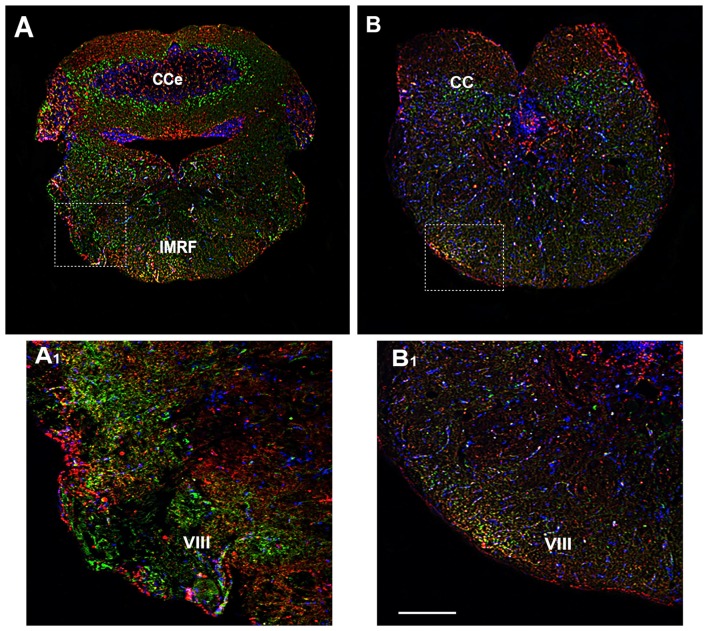
Distribution of OX-2R (red) and CB1R (green), and their co-localization OX-2R/CB1R (yellow) in coronal sections of the rhombencephalon. **(A,B)** OX-2R/CB1R co-expression has been observed in the IMRF and octaval nerve (VIII). **(A_1_,B_1_)** Higher magnification of the fields boxed in **(A,B)** showing the OX-2R/CB1R co-expression in the VIII. DAPI (Blue) was used as a counterstaining to show nuclei. Cce, corpus cerebelli. Scale bar, 250 μm for **(A,B)**; 50 μm for **(A_1_,B_1_)**.

The rhombencephalon is composed of the medulla oblongata, the ventral anterior Pons and the dorso anterior cerebellum (CC) and contributes to several pairs of cranial nerves, such as the octaval nerve (VIII). In the rhombencephalon a corporation of cell groups forms the reticular formation. By immunohistochemical analysis, OX-2R/CB1R co-distribution was observed in the medulla oblongata, in the intermediate reticular formation (IMRF) and in VIII (Figures 7A,A_1_,B,B_1_).

### OX-2R and CB1R Functional Interaction

**Figure 8:**
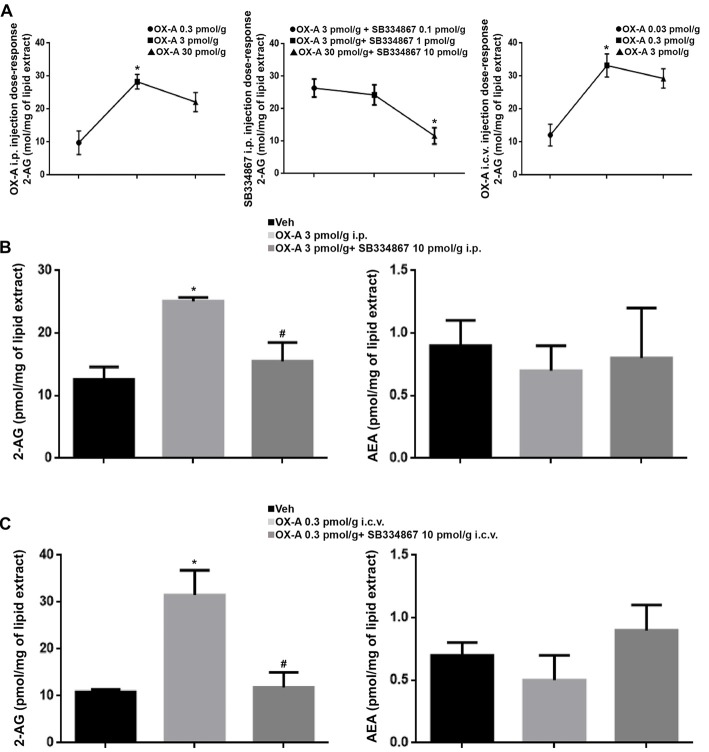
Levels of 2-AG and anandamide (AEA) in the brain of adult zebrafish injected with OX-A and SB334867. **(A)** Dose-response curve of OX-A and SB334867 i.p. injection and OX-A i.c.v. injection. **(B)** 2-AG levels, detected using LC–APCI-MS, significantly increased after i.p. injection with OX-A. The increase was prevented by 30 min of pretreatment with SB334867. No significative differences were found for the AEA. **(C)** 2-AG levels, detected using LC–APCI-MS, significantly increased after i.c.v. injection with OX-A. The increase was prevented by 15 min of pretreatment with SB334867. No significative differences were found for the AEA. Data are means ± SEM. *p < 0.05 vs. ctrl, ^#^*p* < 0.05 vs. OX-A treated.

Several studies have demonstrated that synthetic human OX-A has effective physiological activity in zebrafish and goldfish (Volkoff et al., 1999, 2003; Volkoff and Peter, 2000, 2001; Nakamachi et al., 2006; Yokogawa et al., 2007). A dose-response curve was performed to define the i.p. injected OX-A (OX-A 0.3 pmol/g: 9.8 ± 3.6 pmol/mg, OX-A 3 pmol/g: 28.3 ± 2.2 pmol/mg, OX-A 30 pmol/g 22.1 ± 2.9 pmol/mg), i.c.v. injected OX-A (OX-A 0.03 pmol/g: 12.1 ± 3.3 pmol/mg, OX-A 0.3 pmol/g: 33.2 ± 3.5 pmol/mg, OX-A 3 pmol/g: 29.3 ± 2.9 pmol/mg) and SB334867 (OX-A 3 pmol/g + SB334867 0.1 pmol/g: 26.4 ± 2.8 pmol/mg, OX-A 3 pmol/g + SB334867 1 pmol/g: 24.3 ± 3.1 pmol/mg, OX-A 3 pmol/g + SB334867 10 pmol/g; 11.6 ± 2.5 pmol/mg) effective dose. As shown in the Figure 8, 2-AG levels, detected using LC–APCI-MS, were significantly increased in zebrafish after i.p or i.c.v. injection with OX-A (i.p.: ctrl 12.6 ± 2 pmol/mg vs. OX-A 25.1 ± 0.6 pmol/mg; i.c.v.: ctrl 10.9 ± 0.5 pmol/mg vs. OX-A 31.5 ± 5.3 pmol/mg), and this elevation was prevented by 30 min of pretreatment with the orexin receptor antagonist, SB334867 (i.p.: OX-A 25.1 ± 0.6 pmol/mg vs. SB334867 + OX-A 15.5 ± 3 pmol/mg; i.c.v.: OX-A 31.5 ± 5.3 pmol/mg vs. SB334867 + OX-A 11.8 ± 3.2 pmol/mg). No significative differences were found for the AEA (i.p.: ctrl 0.9 ± 0.2 pmol/mg, OX-A 0.7 ± 0.2 pmol/mg, SB334867 + OX-A 0.8 ± 0.4 pmol/mg; i.c.v.: ctrl 0.7 ± 0.1 pmol/mg, OX-A 0.5 ± 0.2 pmol/mg, SB334867 + OX-A 0.9 ± 0.2 pmol/mg).

## Discussion

In the present study, using immunohistochemistry and confocal imaging, we carried out the neuroanatomical mapping of OX-2R/CB1R co-expression in the whole zebrafish brain. Since the use of a simpler model, which shows the highly conserved structure and functions, could help bridge the lack of data in shorter times, here we have defined a morphological map of OX-2R/CB1R overlapping distributions through the whole zebrafish brain, showing a widespread co-expression of OX-2R and CB1R receptors in numerous brain areas homologous to mammals.

In the telencephalon, OX-2R/CB1R co-expression is intense in the dorsal telencephalic subdivisions Dm and Dl. Such subdivisions are considered as anatomical and functional homologous of the pallial amygdala and part of the hippocampus of mice, respectively (Mueller et al., 2008; Ganz et al., 2014). Behavioral tests after ablation of Dl or Dm also support the idea that these structures perform equivalent functions to hippocampus and amygdala of mammals (Rodríguez et al., 2002; Broglio et al., 2005, 2010; Durán et al., 2010). Indeed, the dorsal telencephalic areas are key regulators of sensory, motor, autonomic and endocrine functions, learning and memory and emotion, like the amygdala and hippocampus of mammals. Moreover, it is known that CB1R and OX receptors are expressed in the basolateral amygdala and in the hippocampus of rodents (Matsuda et al., 1993; Peyron et al., 1998; Tsou et al., 1998; Egertová and Elphick, 2000; Marcus et al., 2001). Therefore, the morphological OX-2R/CB1R overlapping distributions found in the Dm and Dl of zebrafish supports the homologies between orexins/Ecs interaction in the rodent’s homologous brain regions, pointing out their possible involvement in the control of physiological functions regulated by these areas. A similar scenario was found in the subpallium, a region functionally homologous to the mammalian basal ganglia (Cheng et al., 2014), involved in the anxiety-like behaviors, and regulated by orexins and Ecs signaling (Nakamachi et al., 2014; Palotai et al., 2014; Imperatore et al., 2015).

Moving on towards the diencephalon, our morphological analysis showed that several other brain regions are characterized by a widespread OX-2R/CB1R anatomical overlapping distributions. The diencephalon of the zebrafish adult brain includes the thalamus, habenula, PO and hypothalamus, all areas highly conserved across the vertebrates (Stephenson-Jones et al., 2011; Cheng et al., 2014). The anterior and dorsal posterior thalamic nuclei seem to correspond, topologically, to the mammalian rostral and caudal thalamus, respectively. On the other hand, the ventral and dorsal habenular nuclei correspond to the mammalian medial and lateral habenular nuclei, respectively (Amo et al., 2010; Mueller, 2012). One of the most important areas of the zebrafish diencephalon is the PO. This area includes the parvocellular and magnocellular preoptic nuclei, and the SC (Braford and Northcutt, 1983; Wullimann et al., 1996; Rupp and Northcutt, 1998; Filippi et al., 2010; Yamamoto et al., 2010, 2011). The magnocellular preoptic nucleus may represent the brain region homologous to the mammalian paraventricular nucleus (PVN), since several neurons express typical PVN neuropeptides (Forlano and Cone, 2007; Machluf et al., 2011). Remarkably, both the magnocellular preoptic nucleus of zebrafish and PVN of mammals play a pivotal role in the control of homeostasis, although they are located respectively in the PO and hypothalamus (Nieuwenhuys, 2009). In zebrafish, as in all vertebrates, the hypothalamus regulates several physiological and behavioral processes, such as homeostasis, metabolism, energy balance and circadian rhythms (Machluf et al., 2011). Although it has an anatomical organization slightly different from its mammalian counterparts, the hypothalamus of zebrafish contains all the equivalent mammalian hypothalamic cell types located in clusters within the ventral diencephalon (Machluf et al., 2011). The ventral zone of the periventricular hypothalamus in zebrafish is homologous to the hypothalamic arcuate nucleus (Forlano and Cone, 2007; Herget et al., 2014), the main center regulating food intake in mammals. The OX-2R and CB1R anatomical overlapping distributions observed along these diencephalic structures, further reinforce the idea of an orexins/Ecs mediated control of these physiological functions similar in zebrafish and mammals. However, in zebrafish both receptors can be likely expressed in the same or adjacent cell, and future studies can help to elucidate this aspect. Several studies have reported the involvement of orexins/Ecs interactions in the physio-pathological control of homeostasis, food intake and energy balance in mammals (Lau et al., 2017). Indeed, co-localization of these receptors in the hypothalamic area of zebrafish could affect alertness associated to hyperphagic behavior, possibly through the same molecular mechanism described in obese mice, by blunting *Pomc* gene expression and POMC/α-MSH production (Morello et al., 2016).

More caudally, in the diencephalon, OX-2R/CB1R anatomical overlapping distributions has been also observed in other mammalian brain homologous areas. Among these, the PGl and PGm nuclei, which represent the mainly sensory stations projecting to different areas of the pallium and correspond to the mammalian sensory thalamic nuclei (Yamamoto and Ito, 2008; Mueller, 2012).

Finally, TeO, cerebellum and reticular formation are characterized by an intense OX-2R/CB1R co-expression. The TeO, the main visual center located in the mesencephalon of zebrafish, has been defined as homologous to the cortex of amniotes (Kesavan et al., 2017) or to superior colliculus of mammals (Heap et al., 2018), important structures involved in the control of visual functions and movement coordination (Mueller, 2012). The cerebellum and the reticular formation are two main structures of the rhombencephalon, the most evolutionarily and morphologically ancient part of the brain, neuroanatomically and molecularly highly conserved across vertebrates (Gilland and Baker, 1993). The cerebellum is involved in the control of coordinated movement, as in mammals, whereas the corporation of cell groups which form the reticular formation is involved in the control of wakefulness (Moens and Prince, 2002). Therefore, physiological functions, such as wakefulness and locomotion, which are under the control of both, orexins and Ecs signaling, are regulated in zebrafish by brain structures morphologically and functionally highly conserved.

The anatomical OX-2R/CB1R overlapping distributions, observed in numerous zebrafish brain areas, were found to be accompanied by functional OX-2R/CB1R interaction, as revealed by biochemical quantification of the tissue levels of the endocannabinoid 2-AG. At this purpose, we used synthetic human OX-A known to have effective physiological activity in zebrafish and goldfish (Miura et al., 2007; Yokogawa et al., 2007; Facciolo et al., 2009, 2011; Yokobori et al., 2011). We found that the OX-A-mediated OX-2R activation in the brain of adult zebrafish, like in the rodent brain (Ho et al., 2011; Cristino et al., 2013, 2016; Morello et al., 2016), leads to an increase of 2-AG biosynthesis which can be blocked by the antagonism of the OX-2R. Though the SB334867 is not entirely selective for OX receptors and has been reported to be hydrolytically unstable, this compound seems suppress the action of OX-A, suggesting that in zebrafish SB334867 antagonizes orexin at the OX-2R, the only identified orexin receptor in fish (Wong et al., 2011). These findings further support the hypothesis that the orexins/Ecs signaling interaction in the brain of adult zebrafish is similar to that previously found to occur in those regions of the mammalian brain where the two receptors are contiguous. In the murine brain, OX-1R activity releases high levels of 2-AG, which acts as a potent autocrine or paracrine messenger stimulating the same or the neighboring CB1R-expressing cells, respectively (Cristino et al., 2013, 2016, 2017; Imperatore et al., 2016; Morello et al., 2016). The orexins and Ecs signaling interaction in the rodent hypothalamic and extra-hypothalamic areas regulates energy homeostasis (Cristino et al., 2013, 2016, 2017; Morello et al., 2016), nociception (Ho et al., 2011; Cristino et al., 2016, 2017), reward and seeking behaviors (Flores et al., 2013). Moreover, sleep/wake transition, arousal and appetite seem to be affected by changes of OX-A levels accompanied by an increase in 2-AG levels in the same hypothalamic area (Cristino et al., 2013; Kukkonen, 2013; Morello et al., 2016). However, numerous data about the role of orexins/Ecs signaling interaction in the control of physiological functions are still lacking.

Since the assessment of the anatomical distribution of a receptor can provide information on its physiological role, the anatomical map of OX-2R/CB1R overlapping distributions in the whole zebrafish brain, accompanied by the functional interaction of the two receptors, both reported here for the first time, opens the way to the future use of zebrafish as animal models for the analysis of the complex brain functions (including, for example, modulation of appetite, sleep-wake cycle, learning and memory, locomotor activity) regulated by the orexins/Ecs signaling interaction, and of their dysregulation in disease.

## Author Contributions

RI and LD’A designed the experiment, carried out tissue processing, immunohistochemistry, and analyzed the data. OS and HAM designed the experiment and took care of the animal husbandry. FP carried out the lipid extraction and the measurement of 2-AG levels in the brain. PG analyzed the data and wrote the manuscript. LC and EV carried out tissue processing and immunohistochemistry. VM analyzed the data. MP designed the experiment, analyzed the data and wrote the manuscript.

## Conflict of Interest Statement

The authors declare that the research was conducted in the absence of any commercial or financial relationships that could be construed as a potential conflict of interest.
