# Supplementary material for: Overlapping Distribution of Orexin and Endocannabinoid Receptors and Their Functional Interaction in the Brain of Adult Zebrafish
Source: Front Neuroanat. 2018 Jul 30;12:62. doi: 10.3389/fnana.2018.00062 (PMC6077257; doi:10.3389/fnana.2018.00062)
Supplement: Supplementary file 1 [file Data_Sheet_2.docx]

**A**

UserSeq1 14 FTSAQHLNSSADTISHSHAENEDDELLKYIWREYLHPKQYEWVLIAGYILVFLVSLVGNT

UserSeq2 15 WSSASELNETQEPFLNP-TDYDDEEFLRYLWREYLHPKEYEWVLIAGYIIVFVVALIGNV

** ** * * * * ******** ********** ** * * **

UserSeq1 74 LVCFAVWKNHHMRTVTNYFIVNLSFADILVTITCLPASLVVDITETWFFGQTLCKILPYL

UserSeq2 74 LVCVAVWKNHHMRTVTNYFIVNLSLADVLVTITCLPATLVVDITETWFFGQSLCKVIPYL

*** ******************** ** ********* ************* *** ***

UserSeq1 134 QTISVSVSVLTLSCIAQDRWYAICHPLKFKSTAKRARKSIVLIWLVSCIMMIPQAVVMES

UserSeq2 134 QTVSVSVSVLTLSCIALDRWYAICHPLMFKSTAKRARNSIVIIWIVSCIIMIPQAIVMEC

** ************* ********** ********* *** ** **** ***** ***

UserSeq1 194 SSLMPELTNKTSLFTVCDEQWPDEIYPKVYHTCFFIVTYFAPLCLMVLAYIQICHKLWCQ

UserSeq2 194 STVFPGLANKTTLFTVCDERWGGEIYPKMYHICFFLVTYMAPLCLMVLAYLQIFRKLWCR

* * * *** ******* * ***** ** *** *** ********** ** ****

UserSeq1 254 QIPGSSSVLQRQWKSLQCSAHAVGSGESVKVRTSTVSAEAKQVKARRKTARMLMVVLFVF

UserSeq2 254 QIPGTSSVVQRKWKPLQPVSQPRGPGQPTKSRMSAVAAEIKQIRARRKTARMLMVVLLVF

**** *** ** ** ** * * * * * * ** ** ************* **

UserSeq1 314 ALCYLPISILNIMKRVFGAFKNTGNRETVYAWFTFSHWLIYANSAANPIIYNFLSGKFRE

UserSeq2 314 AICYLPISILNVLKRVFGMFAHTEDRETVYAWFTFSHWLVYANSAANPIIYNFLSGKFRE

* ********* ***** * * ************** ********************

UserSeq1 374 EFKAAFICQCSGRGETHKQR-ARGRTSTDSRKSLSTQVNNLDNISRISDQAV

UserSeq2 374 EFKAAFSCCCLGVHHRQEDRLTRGRTSTESRKSLTTQISNFDNISKLSEQVV

****** * * * * ****** ***** ** * **** * * *

UserSeq1: OREXIN RECEPTOR 2 *Danio rerio*

UserSeq2: OREXIN RECEPTOR 2 *Homo sapiens*

**B**

NP_997985.1 MLFPASKSDVKSVLDGVAETTFRTITSGLQYIGSNDIGYDDHIIDGDFSKSGYPLPKPFA

Abcam ------------------------------------------------------------

NP_997985.1 AYRRSSFADKVAPDEELIVKGLPFYPTNSSDVFGNWSHAEDGSLQCGENFMDMECFMILT

Abcam ------------------------------------------------------------

NP_997985.1 PSQQLAIAVLSLTLGTFTVLENLVVLCVILQSRTLRCRPSYHFIGSLAIADLLGSVIFVY

Abcam ------------------------------------------------------------

NP_997985.1 SFLDFHVFHRKDSPNVFLFKLGGVTASFTASVGSLFLTAIDRYVSIHRPLSYRRIVTRTK

Abcam ------------------------------------------------------------

NP_997985.1 AVIAFCMMWAISIIIAVLPLLGWNCKRLNSVCSDIFPLIDENYLMFWIGVTSVLVLFIIY

Abcam ------------------------------------------------------------

NP_997985.1 AYMYILWKAHHHAVRMLRRTSQKSLVVHSADGTKVQTPRPDQARMDIRLAKTLVLILVVL

Abcam ------------------------------------------------------------

NP_997985.1 VICWGPLLAIMVYDLFWRMGDNIKTVFAFCSMLTLLNSTVNPIIYALRSKDLRRAFLAAC

Abcam ------------------------------------------------------------

NP_997985.1 QGCRGTSTTPLQLDNSLESDCHRNQHRAAESCVKTTVKIAKLTMSVSAETSAEAV

Abcam -------------------------------------------MSVSTDTSAEAL

****::*****:

**Suppl. Fig. 2:** Phylogenetic analyses. (**A**) Alignment of aminoacids sequences of OX-2R of Danio rerio (Accession: ABO61386.1 GI: 134142085) and Homo sapiens. 72.6% identity in 412 residues overlap; Score: 1618.0; Gap frequency: 0.5%. (**B**) Alignment of the aminoacid sequence of Danio rerio CB1R (Accession: NP_997985.1 GI: 47086397) and the epitope recognized by the antibody raised against CB1R sequence. Alignements were done by Multiple Sequence Alignment.
