# Supplementary figures and images for: Overlapping Distribution of Orexin and Endocannabinoid Receptors and Their Functional Interaction in the Brain of Adult Zebrafish
Source: Front Neuroanat. 2018 Jul 30;12:62. doi: 10.3389/fnana.2018.00062 (PMC6077257; doi:10.3389/fnana.2018.00062)

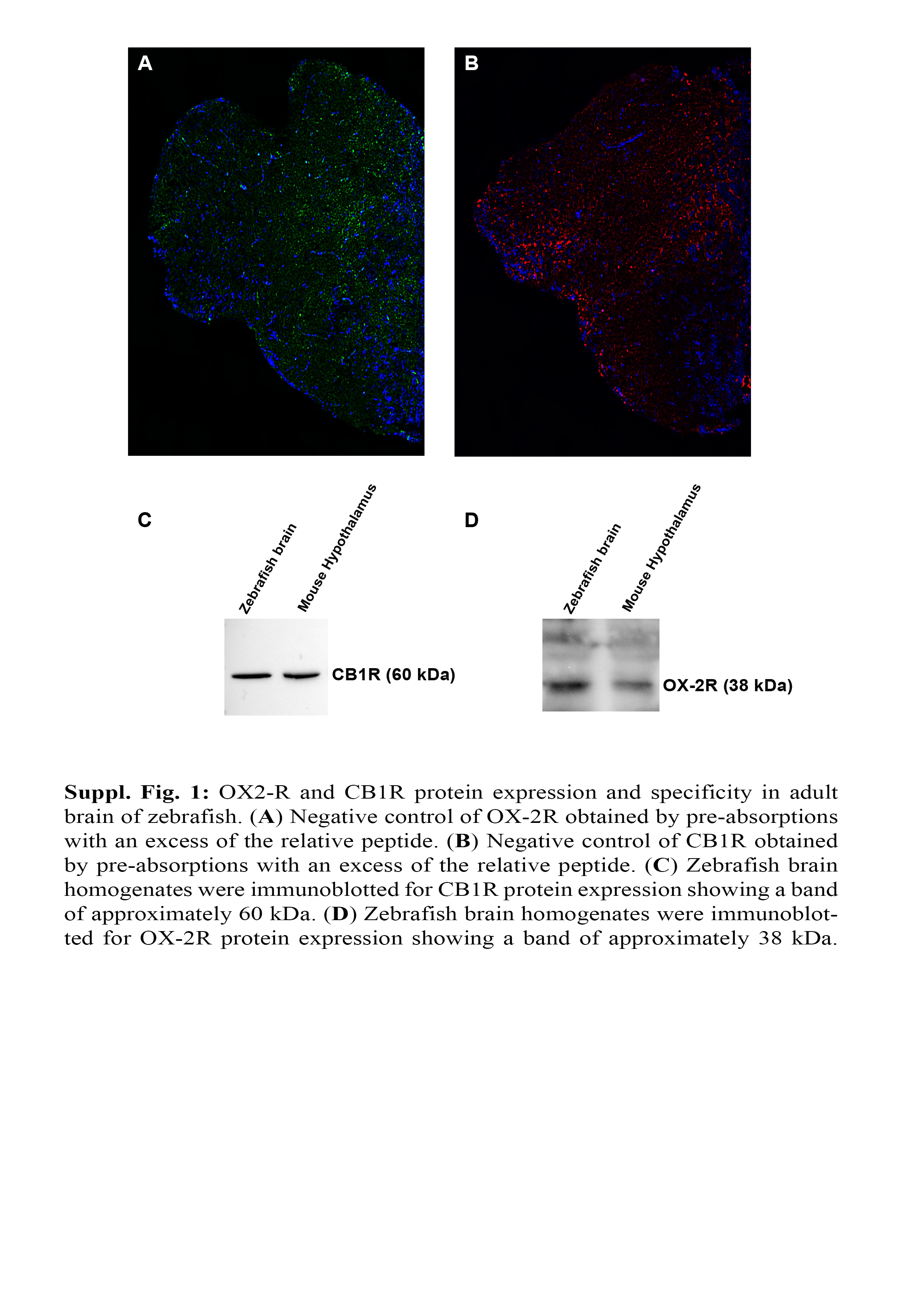

Supplement: Supplementary file 2 [file Image_1.tif]
